# Normative measurements of orbital structures by magnetic resonance imaging

**DOI:** 10.1007/s10792-022-02407-1

**Published:** 2022-07-14

**Authors:** Khizar Rana, Valerie Juniat, Aaron Rayan, Sandy Patel, Dinesh Selva

**Affiliations:** 1 Department of Ophthalmology & Visual Sciences, University of Adelaide, North Terrace, Adelaide, South Australia 5000 Australia; 2 South Australian Institute of Ophthalmology, Royal Adelaide Hospital, Port Road, Adelaide, South Australia 5000 Australia; 3 Department of Medical Imaging, Royal Adelaide Hospital, Port Road, Adelaide, South Australia 5000 Australia

**Keywords:** Orbit, Extraocular muscle, Superior ophthalmic vein, Magnetic resonance imaging

## Abstract

**Purpose:**

We describe and compare the normative values of orbital structures in an Australian cohort on *T*1-weighted MRI and fat-suppressed contrast-enhanced *T*1-weighted MRI.

**Methods:**

Retrospective review of patients who underwent 3*T* orbital MRI. The maximum extraocular muscle (EOM) and superior ophthalmic vein (SOV) diameters on normal orbits were recorded. The extraocular muscle diameters were summed to produce the sum of all muscles.

**Results:**

The normal measurements (mean ± SD) from 141 orbits that had fat-suppressed contrast-enhanced MRI: medial rectus, 4.1 ± 0.5 mm; lateral rectus (LR), 3.9 ± 0.7 mm; superior muscle group (SMG), 4.5 ± 0.7 mm; inferior rectus (IR), 4.6 ± 0.7 mm; and SOV, 1.8 ± 0.7 mm. The normal measurement from 84 orbits that had *T*1-weighted MRI: MR, 4.1 ± 0.5 mm; LR, 3.4 ± 0.6 mm; SMG, 4.3 ± 0.7 mm; IR, 4.6 ± 0.7 mm; SOV, 2.0 ± 0.7 mm. Eighty-four orbits had both MRI sequences performed. The LR, SMG and the sum of all muscles were significantly larger on fat-suppressed contrast-enhanced *T*1-weighted MRI sequence than the *T*1-weighted sequence (*P* < 0.01), whereas the SOV was significantly larger on the *T*1-weighted sequence (*P* < 0.01).

**Conclusion:**

These data may aid in diagnosing pathological enlargement of the EOMs and SOV on different scan sequences.

## Introduction

Enlargement of the orbital structures including the extraocular muscles (EOMs), optic nerve sheath (ONSD) and the superior ophthalmic vein (SOV) can be seen in a range of orbital inflammatory, neoplastic, and vascular conditions. An understanding of the normal diameters of orbital structures can aid in diagnosing enlargement.

Fat-suppressed contrast-enhanced *T*1-weighted MRI is the preferred modality for the evaluation of orbital pathology [1]. The contrast can help highlight areas of inflammation or neoplasia that are otherwise difficult to assess. Previous studies have however only reported normative data on *T*1-weighted MRI studies [2–4], and it is likely to vary with different scan sequences.

In the current study, we characterise and compare the normative values of orbital structures on *T*1-weighted MRI and fat-suppressed contrast-enhanced *T*1-weighted MRI and investigate how these may be affected by age and gender.

## Methods

### Subjects

A retrospective review of patients who underwent magnetic resonance imaging (MRI) orbit studies for suspected orbital disease. Patients with diseases known to affect bilateral orbits (e.g. thyroid eye disease, IgG4 disease and trauma), previous orbital surgery, or poor scan quality were excluded. In patients with a unilateral orbital lesion, only the normal side was used. The study was approved by the Central Adelaide Local Health Network ethics committee.

### MRI examination

All patients were evaluated using Magnetom 3T Skyra scanner (Siemens AG, Munich, Germany) with a turbo spin-echo sequence (TR/TE, 500/15; field of view, 200 × 200 mm; matrix, 512 × 512; slice thickness 3 mm). Contrast enhanced images were obtained after intravenous administration of a standard weight-based dose of gadolinium. Patients were asked to maintain forward gaze and gentle eye closure to prevent asymmetric extraocular muscle contraction. Axial scans were obtained parallel to the optic nerve. Coronal scans were perpendicular to the axial plane.

### Image analysis

**Fig. 1:**
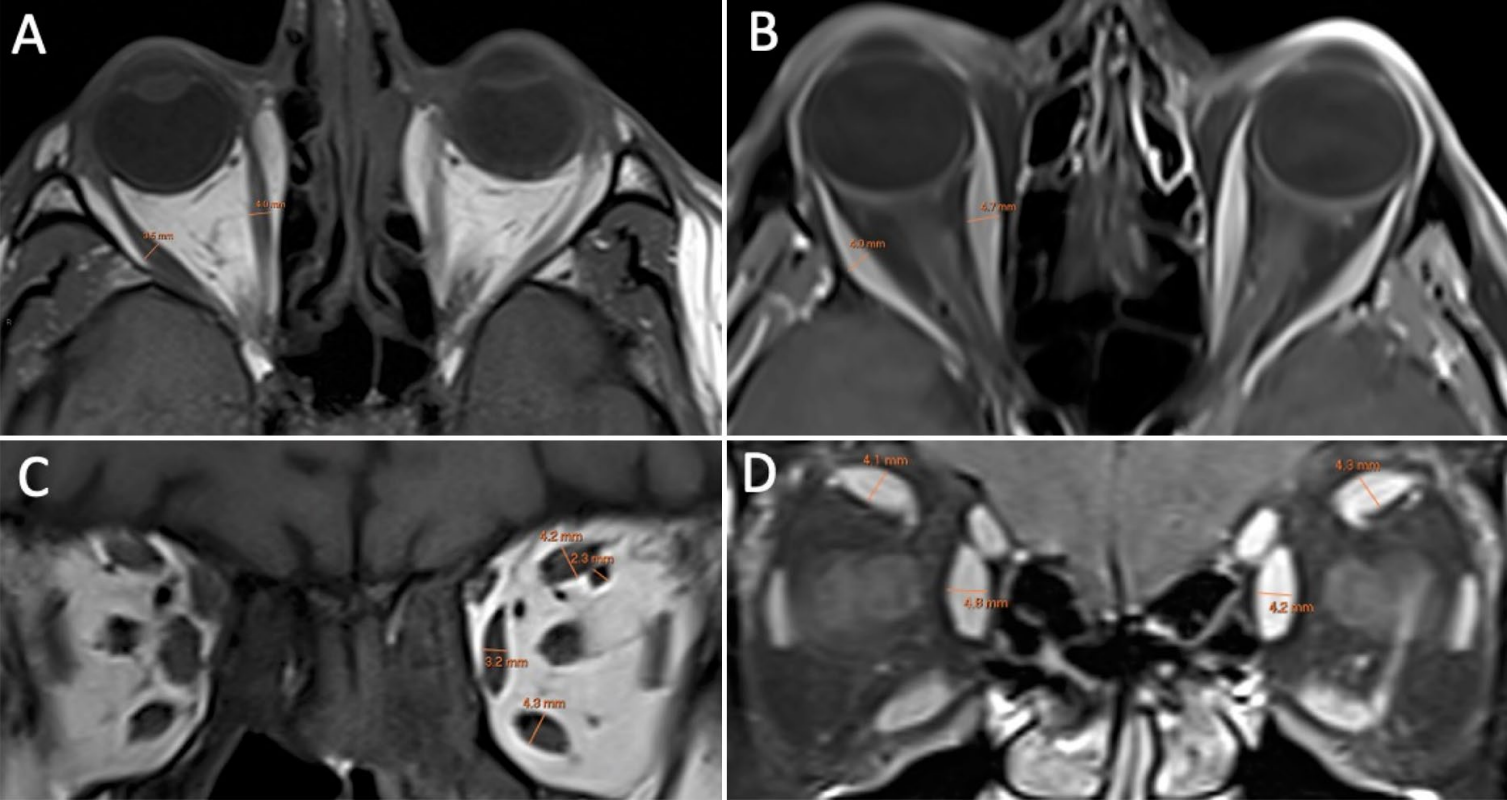
Axial T1-weighted MRI (A) and fat-suppressed contrast enhanced T1-weighted MRI (B) showing the measurements of the right medial and lateral rectus muscles perpendicular to the muscle belly. Coronal T1-weighted MRI (C) showing the measurements of the superior muscle group, medial rectus, inferior rectus and superior ophthalmic vein. Fat-suppressed contrast enhanced T1-weighted MRI (D) showing the measurements of the medial rectus and superior muscle group

**Fig. 2:**
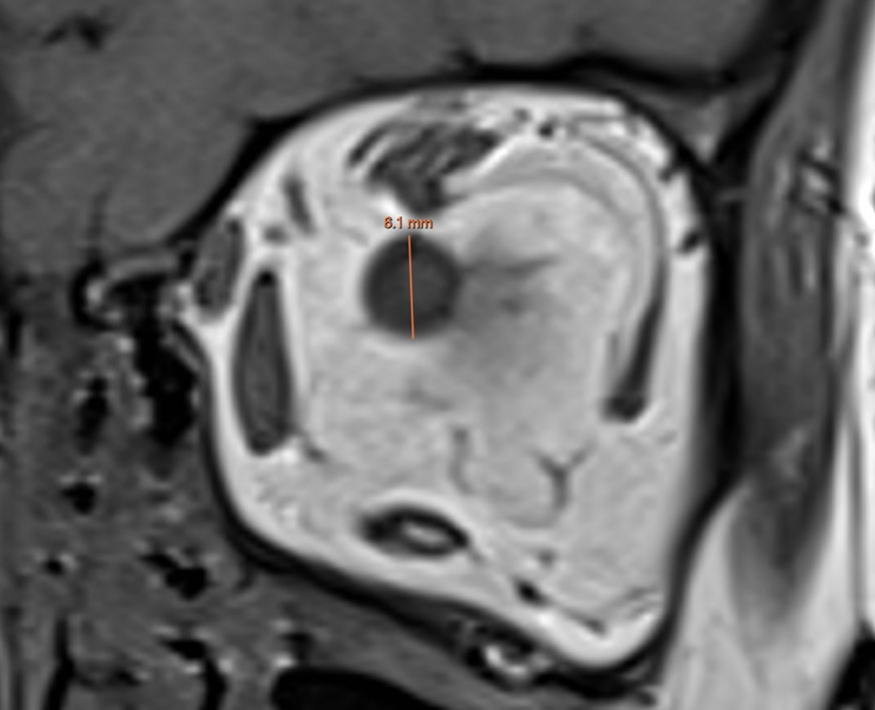
Coronal T1-weighted MRI showing the optic nerve sheath diameter measurement

Extraocular muscle thickness of the medial and lateral recti was measured on axial scans perpendicular to the muscle belly (Fig. 1A, B). The superior rectus and levator palpebrae superioris could not be reliably distinguished and were considered as the superior muscle group. Coronal scans were used to measure the maximum diameters of the superior muscle group, medial and inferior recti, and the SOV (Fig. 1C, D). The ONSD was also measured on coronal *T*1-weighted scans (Fig 2). The measurements were taken perpendicular to the long axis of these structures at their maximum diameters. All measurements were taken on high resolution picture archiving and communication system (PACS).

### Statistical analysis

All statistical analysis was performed using Stata 13.0 (StataCorp, College Station, Texas). The mean values of orbital structures were calculated. Data were presented as mean ± standard deviation. In patients who had both orbits examined, only the right orbit was used. The diameters of the superior muscle group, medial, lateral and inferior recti were summed to a total of all muscles in any individual. The ratio of the diameter of the medial rectus on a coronal scan to that measured on axial scan was calculated. The independent samples *t*-test was used to compare the data from male and female patients. Pearson’s correlation coefficient was used to assess the correlation between age and orbital structures. The paired t-test was used to compare the diameters from the two different MRI sequences. To assess for inter and intraobserver reliability, thirty scans were assessed by a second reviewer (AR) and fifteen scans were reassessed by the first reviewer (KR). The reviewers were blinded to the original results and the intraclass correlation coefficient (ICC) was determined. For all statistical analyses, a *P*-value less than 0.05 was considered significant.

## Results

### Fat-suppressed contrast-enhanced *T*1-weighted MRI

Data are reported from 141 orbits from 141 patients (64 male, 77 female). The mean age of these participants was 58 ± 18 years (20–94 years). The mean diameters and standard deviations of the EOMs and SOV for all participants, as well as male and female groups, are given in Table 1. The mean ratio of the diameter of the medial rectus measured on coronal plane to the diameter measured on axial plane was 0.995 (95% CI, 0.82–1.32).

**Table 1:** Normative orbital measurements on fat suppressed contrast-enhanced *T*1-weighted MRI

Measurement	Plane	Total (mean ± SD in mm)	Male (mean ± SD in mm)	Female (mean ± SD in mm)	*P*-value
LR	Axial	3.91 ± 0.70	4.11 ± 0.63	3.74 ± 0.72	< 0.01
MR	Axial	4.12 ± 0.53	4.06 ± 0.51	4.17 ± 0.54	0.22
MR	Coronal	4.09 ± 0.55	4.08 ± 0.55	4.10 ± 0.55	0.77
SMG	Coronal	4.49 ± 0.69	4.62 ± 0.68	4.38 ± 0.69	0.04
IR	Coronal	4.59 ± 0.72	4.71 ± 0.72	4.49 ± 0.71	0.08
SOV	Coronal	1.79 ± 0.67	1.80 ± 0.75	1.78 ± 0.60	0.87
Sum of muscles	Axial, coronal	17.1 ± 1.8	17.5 ± 1.67	16.8 ± 1.88	0.02

*LR* lateral rectus, *MR* medial rectus, *SMG* superior muscle group, *IR* inferior rectus, *SOV* superior ophthalmic vein

Males had significantly larger diameters of the lateral rectus, superior muscle group and sum of all muscles (*p* < 0.05). No significant differences were seen for the other EOMs or SOV. Significant positive correlation was seen between age and lateral rectus (*r* = 0.19, *p* = 0.02) and negative correlation with medial rectus (*r* = − 0.24, *p* < 0.01). The measurements of orbital structures across different age groups is given in Table 2.

**Table 2:** Descriptive statistics in millimetres of orbital structures according to age on fat suppressed contrast-enhanced *T*1-weighted MRI

Measurement	Plane	20–39	40–59	60–79	80–99
LR	Axial	3.6	3.9	4.1	3.8
MR	Axial	4.2	4.3	4.0	3.9
MR	Coronal	4.2	4.2	4.1	3.7
SMG	Coronal	4.3	4.6	4.5	4.5
IR	Coronal	4.4	4.6	4.6	4.8
SOV	Coronal	1.7	1.7	1.9	1.7
Sum of muscles	Axial, coronal	16.5	17.3	17.3	17.0

*LR* lateral rectus, *MR* medial rectus, *SMG* superior muscle group, *IR* inferior rectus, *SOV* superior ophthalmic vein

### *T*1-weighted MRI

Data are reported from 84 orbits from 84 patients (40 male, 44 female). The mean age of these participants was 56 ± 18 years (20–87 years). The mean diameters and standard deviations of the EOMs and SOV are given in Table 3. The mean ratio of the diameter of the medial rectus measured on coronal plane to the diameter measured on axial plane was 0.98 (95% CI, 0.73–1.17).

**Table 3:** Normative orbital measurements on *T*1-weighted MRI

Measurement	Plane	Total (mean ± SD in mm)	Male (mean ± SD in mm)	Female (mean ± SD in mm)	*P*-value
LR	Axial	3.44 ± 0.61	3.61 ± 0.53	3.30 ± 0.65	0.02
MR	Axial	4.12 ± 0.52	4.11 ± 0.50	4.12 ± 0.55	0.93
MR	Coronal	4.01 ± 0.53	4.04 ± 0.53	3.98 ± 0.53	0.63
SMG	Coronal	4.29 ± 0.67	4.36 ± 0.59	4.23 ± 0.74	0.39
IR	Coronal	4.62 ± 0.73	4.68 ± 0.73	4.57 ± 0.73	0.48
ONSD	Coronal	5.72 ± 0.85	5.79 ± 0.98	5.75 ± 0.74	0.84
SOV	Coronal	1.96 ± 0.71	1.92 ± 0.67	2.00 ± 0.75	0.59
Sum of muscles	Axial, coronal	16.5 ± 1.73	16.8 ± 1.53	16.2 ± 1.88	0.15

*LR* lateral rectus, *MR* medial rectus, *SMG* superior muscle group, *IR* inferior rectus, *ONSD* optic nerve sheath diameter, *SOV* superior ophthalmic vein

Males had a significantly larger diameter of the lateral rectus than females (*p* < 0.05). No significant differences were seen for the other EOMs, ONSD or SOV. There was significant negative correlation between age and the medial rectus on a coronal plane (*r* = − 0.25, *p* = 0.02). The measurements of orbital structures across different age groups is given in Table 4.

**Table 4:** Descriptive statistics in millimetres of orbital structures according to age on *T*1-weighted MRI

Measurement	Plane	20–39	40–59	60–79	80–99
LR	Axial	3.2	3.4	3.6	3.4
MR	Axial	4.2	4.1	4.1	4.2
MR	Coronal	4.2	4.1	3.9	3.9
SMG	Coronal	4.3	4.3	4.3	4.4
IR	Coronal	4.7	4.5	4.7	4.5
SOV	Coronal	1.6	2.0	2.1	1.7
Sum of muscles	Axial, coronal	16.4	16.3	16.6	16.5

*LR* lateral rectus, *MR* medial rectus, *SMG* superior muscle group, *IR* inferior rectus, *SOV* superior ophthalmic vein

### Fat-suppressed contrast-enhanced *T*1-weighted MRI versus *T*1-weighted MRI

Data are reported from 71 orbits from 71 patients (34 male, 37 female) who had both fat-supressed contrast enhanced *T*1-weighted MRI and *T*1-weighted MRI. The mean age of these participants was 58 ± 17 years (20–87 years). The mean EOM and SOV diameters are detailed in Table 5. The lateral rectus, superior muscle group and the sum of all muscles were significantly larger on fat-suppressed contrast-enhanced *T*1-weighted MRI studies than the *T*1-weighted studies (*p* < 0.01). The SOV was significantly larger on *T*1-weighted studies as compared to the fat-suppressed contrast-enhanced *T*1-weighted MRI (*p* < 0.01).

**Table 5:** Normative orbital measurements on fat suppressed contrast-enhanced *T*1-weighted MRI compared to *T*1-weighted MRI

Measurement	Plane	*T*1 MRI (mean ± SD in mm)	*T*1 FS CE (mean ± SD in mm)	*P*-value
LR	Axial	3.46 ± 0.64	3.87 ± 0.67	< 0.01
MR	Axial	4.13 ± 0.53	4.10 ± 0.49	0.54
MR	Coronal	4.01 ± 0.52	4.04 ± 0.50	0.48
SMG	Coronal	4.30 ± 0.68	4.51 ± 0.66	< 0.01
IR	Coronal	4.70 ± 0.71	4.59 ± 0.73	0.07
SOV	Coronal	2.13 ± 0.72	1.89 ± 0.72	< 0.01
Sum of muscles	Axial, coronal	16.6 ± 1.74	17.1 ± 1.71	< 0.01

*LR* lateral rectus, *MR* medial rectus, *SMG* superior muscle group, *IR* inferior rectus, *SOV* superior ophthalmic vein

Intraobserver reliability for the EOMs and SOV was excellent (ICC 0.87–0.97) and interobserver reliability was good to excellent (ICC 0.71–0.86). The normal ranges of the EOM and SOV diameter on *T*1-weighted imaging across different studies are given in Tables 6 and 7.

## Discussion

We evaluated the normative orbital structures using high field (3*T*) *T*1-weighted MRI and fat-suppressed contrast-enhanced *T*1-weighted MRI. Previous studies have only reported data using standard *T*1-weighted MRI [3, 4].

Extraocular muscle enlargement may be diagnosed when the muscle diameter exceeds the normal range, defined as the value two standard deviations above the normal population mean (Table 6). Diameters of more than 5 mm for the horizontal recti and more than 6 mm for the superior muscle group or inferior rectus may be considered enlarged. Ethnic differences in normal anatomical structures may explain the larger muscle diameters reported by Shen, Fong, Wong, Looi, Chan, Rootman and Seah [4].

Age and gender may impact the normal dimensions of orbital structures. Our study found that males had a larger diameter of the lateral rectus and superior muscle group on fat-suppressed contrast-enhanced *T*1-weighted MRI and a larger lateral rectus on a *T*1-weighted MRI. This is in-line with previous studies which have also shown males to have significantly larger muscle diameters [3, 5]. We also found a significant positive correlation between age and lateral rectus diameter and a negative correlation between age and the medial rectus diameter. Ozgen and Aydingöz [3] also found a significant positive correlation between age and the lateral rectus diameter.

We found some differences in the measurements obtained from the two MRI sequences. The lateral rectus and the superior muscles group were significantly larger on the fat-suppressed contrast-enhanced *T*1-weighted MRI, whereas the SOV diameter was smaller. This is likely due to partial volume averaging at the muscle-fat interface. On a fat-suppressed image, the muscle signal dominates within the voxels at the muscle-fat interface whereas the fat signal dominates at the muscle-fat interface on a standard *T*1-weighted scan. In diagnosing EOM or SOV enlargement, the normative values derived from the same MRI sequence should be used.

We found the SOV diameter to be larger on a *T*1-weighted MRI scan as compared to a fat-suppressed contrast-enhanced *T*1-weighted MRI. Our reported SOV mean diameter of approximately 2.0 mm is similar to previously reported data (Table 7) [3, 6, 7]. Adam et al. [8] reviewed 113 cases of a dilated SOV and used a cut-off value of greater than 3.0 mm on two contiguous coronal slices to define a dilated SOV. This may be a reasonable cut-off value for patients with symptomatic orbital disease. It should be noted that there may be considerable physiological fluctuation in the size of the SOV (as we see in venous malformations at different imaging time points) due to fluctuations in regional venous pressures. This may account for some of the variation in SOV measurement between sequences in a given patient.

Optic nerve sheath complex enlargement may arise from primary tumour, inflammation, metastatic tumour or increased intracranial pressure. Shen, Fong, Wong, Looi, Chan, Rootman and Seah [4] reported the ONSD at two preselected planes at 0 mm and 7 mm posterior to the globe. The mean diameter at 0 mm behind the globe was a 5.4 mm with a normal range of 4.0–6.8 mm. We measured the ONSD at its maximum coronal diameter and found a mean diameter of 5.7 mm with a normal range of 4.0–7.4 mm (mean ± 2 SD).

Studies on normative orbital structures have used different scan sequences and planes for measuring the EOMs and ONSD [4, 6]. A consistent protocol is required to make meaningful comparisons between studies. As it is the enlargement of structures that is of most clinical relevance, measuring orbital structures at their maximum diameters is of most utility and has been done in the vast majority of studies [2, 3]. Measurements conducted at preselected planes posterior to the globe may not capture the maximum diameters of these structures [4]. They may also be difficult to replicate with different imaging protocols. We suggest measuring the maximum muscle diameters and using an axial plane for the measurements of the medial and lateral recti and a coronal plane for the superior muscle group, inferior rectus, ONSD and SOV. (Tables 6 and 7).

**Table 6:** Normative ranges of extraocular muscles on *T*1-weighted MRI

Study	Lateral rectus		Medial rectus		Superior muscle group		Inferior rectus	
	Mean (mm)	Range (± 2 SD)	Mean (mm)	Range (± 2 SD)	Mean (mm)	Range (± 2 SD)	Mean (mm)	Range (± 2 SD)
Our study	3.4	2.2–4.7	4.1	3.1–5.2	4.3	3.0–5.6	4.6	3.2–6.1
Ozgen et al. [3]	3.7	2.6–4.8	4.0	3.2–4.9	4.4	3.1–5.6	4.8	3.7–6.0
Shen et al. [4]	4.5	2.7–6.3	5.1	3.3–6.9	4.8	2.6–7.0	5.4	3.4–7.4

**Table 7:** Superior ophthalmic vein diameters on *T*1-weighted MRI and CT

Study	Mean (mm)	Range (mm)	MRI/CT
Our study	1.96	0.5–3.4^†^	MRI
Ozgen et al. [3]	1.9	1.0–2.9^†^	MRI
Brightbill et al. [7]	2.2	1.4–3.6^‡^	CT

^†^mean ± 2 SD

^‡^Minimum to maximum

Additionally, we measured the medial rectus on both an axial and coronal plane with no significant difference being seen. This is in-line with a previous CT study which also found no significant difference for the medial rectus diameter on an axial plane as compared to a coronal plane [5]. The coronal measurement of the medial rectus may be a suitable alternative in cases where axial views are not available. However, the lateral rectus cannot be reliably measured on a coronal plane as its oblique course can result in an overestimation of its diameter [5, 9].

In summary, we present 3*T*-MRI derived normative orbital data on *T*1-weighted MRI and fat-suppressed contrast-enhanced *T*1-weighted MRI. Normative values of some orbital structures may vary according to the scan sequence that is used. This data may help clinicians to diagnose enlargement of the EOMs, SOV or ONSD.
